# Metagenome-Assembled Genome of a Novel *Cyclobacteriaceae* Epibiont of a Heterotrophic Diazotroph

**DOI:** 10.1128/mra.00594-22

**Published:** 2023-01-23

**Authors:** Michelle DeMers, Elaina D. Graham, John F. Heidelberg, Eric A. Webb

**Affiliations:** a Department of Biological Sciences, University of Southern California, Los Angeles, California, USA; Montana State University

## Abstract

Here, we describe the metagenome-assembled genome (MAG) HetDA_MAG_SS2, in the family *Cyclobacteriaceae*. It was found in association with a HetDA cyanobiont isolated from a Station ALOHA *Trichodesmium* colony. Annotation suggests that HetDA_MAG_SS2 is a chemoorganoheterotroph with the potential for lithoheterotrophy, containing genes for aerobic respiration, mixed acid fermentation, dissimilatory nitrate reduction to ammonium, and sulfide oxidation.

## ANNOUNCEMENT

Here, we describe a novel cyanobacterium-associated epibiont, HetDA_MAG_SS2. *Trichodesmium* colonies were picked from the oligotrophic North Pacific Ocean near Station ALOHA, as detailed by Momper et al. (1), and transferred to sterile YBC-II medium at 24°C with a 12-h light (100 μmol photons m^−2^ s^−1^)/12-h dark cycle. The enrichment was grown for 5 years prior to sequencing and was transferred to fresh medium every month. A 50-mL subsample was gravity filtered onto a 5.0-μm polycarbonate filter, and DNA was extracted using the Qiagen DNeasy PowerSoil kit. The DNA was sent to the University of Southern California Epigenome Center and sequenced on an Illumina MiSeq sequencer (paired-end 250-bp reads, with a total of 4725,335 raw reads) using the MiSeq reagent kit v2 with 300 cycles. Reads were trimmed using Trimmomatic v0.38 (parameters: –phred33, ILLUMINACLIP:TruSeq3-PE.fa:2:30:10 SLIDINGWINDOW:10:28 MINLEN:50) (2). Raw reads were subsampled at the following percentages: 1, 3, 5, 10, 25, 30, and 50. Subsampled reads were assembled using MEGAHIT v1.2.8 (3). As described by Tully et al. (4), primary contigs from initial MEGAHIT runs were passed through CD-HIT-EST v4.8.1 (parameters: –M 500000 –c 0.99 –n 10) to identify overlapping primary contigs (5), followed by a secondary assembly in Minimus2 (AMOS v3.1.0) (parameters: –D OVERLAP=1000 MINID=98) (6). The Minimus2-generated contigs and the primary contigs that did not assemble with Minimus2 were combined to create the final contigs. The reads were mapped to the resulting assembly using Bowtie2 v2.3.5 (7) and filtered using CoverM v0.4.0 (parameters: –min-read-percent-identity 0.95 –min-read-aligned-percent 0.75) (8) to calculate coverage. The assembly was binned using MetaBat2 v2.12.1 (9), BinSanity-wf v0.3.8 (10), and Concoct v1.1.0 (11) using default parameters for each, and DASTool v1.1.2 (12) was used to determine a final set of metagenome-assembled genomes (MAGs). All bins were run through MetaSanity v1.3.0 (13), PhyloSanity, and FuncSanity for phylogeny and functional annotation using the default configuration.

The assembled genome of HetDA_MAG_SS2 was identified as novel using GTDB-tk-calculated relative evolutionary divergence (RED) scores (14) and was 5,472,162 bp in length and 97% complete, as determined by CheckM (15). There were 339 contigs, with an *N*_50_ value of 2,360 bp, a GC content of 42.8%, and a coding density of 92.35%. HetDA_MAG_SS2 is a member of the phylum *Bacteroidota*, falling into the family *Cyclobacteriaceae*. The closest genome representative was UBA7330 (GenBank assembly accession number GCA_002471275.1), which was only 85.53% similar, based on the average nucleotide identity (ANI) derived from GTDB-tk.

Using KEGG (16) annotations, it was found that HetDA_MAG_SS2 contains the genes necessary for aerobic respiration, including glycolysis and the tricarboxylic acid (TCA) cycle. In addition, it contains the *cbb*_3_-type cytochrome *c* oxidase and the cytochrome *bd* complex, which are necessary for respiration in microaerobic environments (17). This finding, paired with the presence of genes for mixed acid (e.g., lactate, acetate, and ethanol) fermentation, indicate that this may be a facultative aerobe.

HetDA_MAG_SS2 contains the genes for sulfide oxidation (KEGG Orthology codes K17218 and K17229) and sulfur assimilation (KEGG Orthology codes K00392, K00380, and K00381), which suggests that it may can use sulfur compounds for both energy and biosynthesis (18). It also contains genes for dissimilatory nitrate reduction to ammonium (DNRA) and nitrous oxide reduction and the *nosZ* gene (KEGG Orthology code K00376) for nitrous oxide reduction to N_2_ (19–21).

In summary, HetDA_MAG_SS2 is a chemoheterotroph with potential roles in both sulfur and nitrogen cycling.

### Data availability.

Raw sequences and MAGs are available under BioProject accession number PRJNA719568. Raw reads are available in the SRA under accession number SRR14140256. The genome is available under BioSample accession number SAMN18613317.
